# AI-Enabled Framework for Fog Computing Driven E-Healthcare Applications

**DOI:** 10.3390/s21238039

**Published:** 2021-12-01

**Authors:** Ali Hassan Sodhro, Noman Zahid

**Affiliations:** 1 Department of Computer Science, Kristianstad University, SE-291 88 Kristianstad, Sweden; 2 Shenzhen Institutes of Advanced Technology, Chinese Academy of Sciences, Shenzhen 518000, China; 3 Office of Research, Innovation and Commercialization (ORIC), The University of Faisalabad, Faisalabad 37610, Punjab, Pakistan; noman.mece17@iba-suk.edu.pk

**Keywords:** 6G, AI, fog computing, e-health, cyber physical system, interoperability, analytic hierarchy process

## Abstract

Artificial Intelligence (AI) is the revolutionary paradigm to empower sixth generation (6G) edge computing based e-healthcare for everyone. Thus, this research aims to promote an AI-based cost-effective and efficient healthcare application. The cyber physical system (CPS) is a key player in the internet world where humans and their personal devices such as cell phones, laptops, wearables, etc., facilitate the healthcare environment. The data extracting, examining and monitoring strategies from sensors and actuators in the entire medical landscape are facilitated by cloud-enabled technologies for absorbing and accepting the entire emerging wave of revolution. The efficient and accurate examination of voluminous data from the sensor devices poses restrictions in terms of bandwidth, delay and energy. Due to the heterogeneous nature of the Internet of Medical Things (IoMT), the driven healthcare system must be smart, interoperable, convergent, and reliable to provide pervasive and cost-effective healthcare platforms. Unfortunately, because of higher power consumption and lesser packet delivery rate, achieving interoperable, convergent, and reliable transmission is challenging in connected healthcare. In such a scenario, this paper has fourfold major contributions. The first contribution is the development of a single chip wearable electrocardiogram (ECG) with the support of an analog front end (AFE) chip model (i.e., ADS1292R) for gathering the ECG data to examine the health status of elderly or chronic patients with the IoT-based cyber physical system (CPS). The second proposes a fuzzy-based sustainable, interoperable, and reliable algorithm (FSIRA), which is an intelligent and self-adaptive decision-making approach to prioritize emergency and critical patients in association with the selected parameters for improving healthcare quality at reasonable costs. The third is the proposal of a specific cloud-based architecture for mobile and connected healthcare. The fourth is the identification of the right balance between reliability, packet loss ratio, convergence, latency, interoperability, and throughput to support an adaptive IoMT driven connected healthcare. It is examined and observed that our proposed approaches outperform the conventional techniques by providing high reliability, high convergence, interoperability, and a better foundation to analyze and interpret the accuracy in systems from a medical health aspect. As for the IoMT, an enabled healthcare cloud is the key ingredient on which to focus, as it also faces the big hurdle of less bandwidth, more delay and energy drain. Thus, we propose the mathematical trade-offs between bandwidth, interoperability, reliability, delay, and energy dissipation for IoMT-oriented smart healthcare over a 6G platform.

## 1. Introduction

The 6G communication technology has become the center of attention of several researchers due to its amazing and empowering traits in various domains, revealed through impressive revolutionary progress in most of the areas, and further is projected to be evident from 2030 onward [1]. Numerous countries have already launched interesting projects related to 6G, for instance, Finland, the USA, China, South Korea, and Japan from 2018 to date [2,3,4,5,6]. In addition, many scientific and technical approaches and significant contributions have been made by researchers regarding 6G throughout the world [7]. This is all due to the challenges and less mature platform of 5G towards modern living styles, including, for example, societal and business needs such as being less supportive to holographic communication at lower data rates, and to intelligent monitoring and provisioning of the patient’s well-being [8]. 

**Figure 1:**
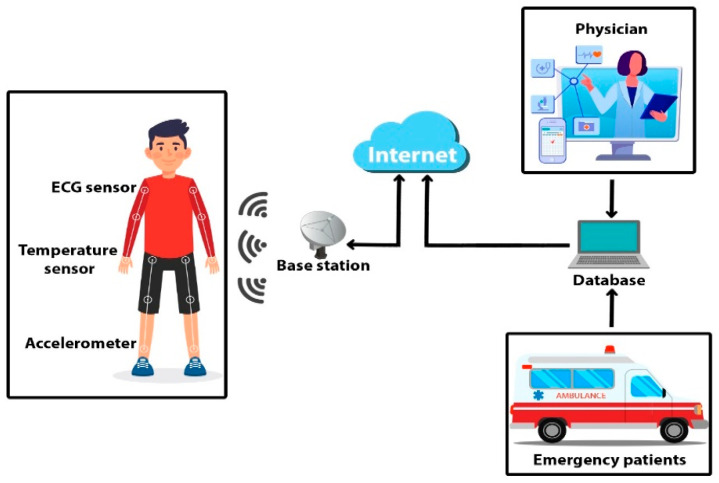
Architecture of IoT-based smart healthcare applications.

Artificial Intelligence (AI) and 6G are playing the major role in fairly allocating the resources in sensitive and precious healthcare scenarios. The Internet of Things (IoT) has remarkably revolutionized the healthcare market with the help of sensors, actuators, connectors and so on which leads toward the cyber physical system (CPS) [9,10]. The emerging internet paradigm is not sufficient to cover most of the application range due to its centralized behavior. Therefore, to bridge that gap the new notion named the Internet of Medical Things (IoMT) was introduced, where end-users and their personal entities are cornerstones in the fast and emerging internet world [3]. There exists a revolution in emerging technologies such as 5G, edge/cloud computing, Bluetooth low-power energy (BLE), and interconnected devices of the IoMT [4]. The collected ECG signals of elderly emergency patients are transmitted to electronic health medical theaters for entertaining every patient [5]. Breakthroughs in wearable devices, physiological signal collection, medical information transmission through sensors and low-power integrated circuits have encouraged and given a new shape and direction to smart healthcare [6,7,8], as shown in Figure 1. 

**Figure 2:**
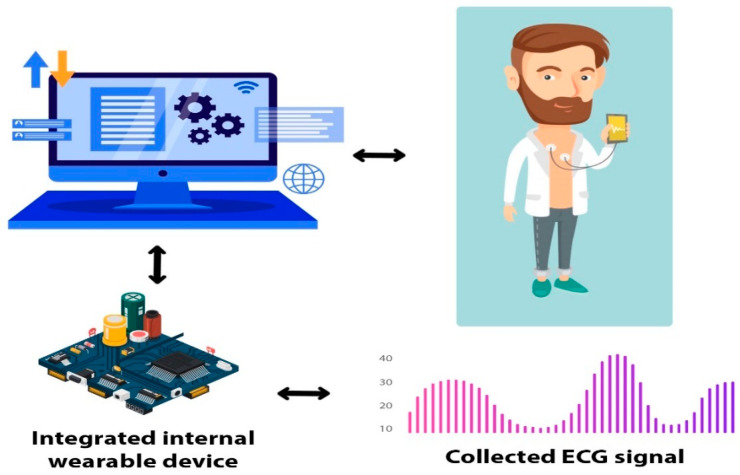
Wearable ECG data collection hardware platform.

The IoT has refined the CPS and is considered as one of the emerging applications for healthcare platforms. CPS can be described as the system of miniaturized sensors in close contiguity to a person’s body that coordinate individually for facilitating each patient [9,10]. However, there are also other exciting and charming challenges to be faced, such as the variability of the wireless channel determined by the changes of body posture [11,12]. Medical inheritance of CPS-based smart healthcare has quickly begun to lead the way in association with the IoT and other emerging technologies, i.e., Bluetooth low-power energy (BLE) [13,14]. However, the body area network is adaptable with various attractive applications in addition to body sensor networks. Although WBSN devices clarify the requirements by restricting themselves to a much smaller range (i.e., from 0.01 to about 2.00 m) [15,16], this short range allows developers to take benefit from various aspects of the human body; for example, the human body can itself become a channel confining the needs of conventional antennas [17,18,19,20]. Avoiding the demand of an extra antenna, the power consumption of WBSN devices can be shrunk to 0.1–1.0 mW. This reduced amount of power can be obtained directly through a harvesting mechanism, drastically reducing the need for traditional power sources, such as batteries [21,22,23,24]. In addition, AI-based cloud-computing platforms synchronize the processes of heterogenous platforms by developing sustainable, reliable, convergent, and interoperable networks, as shown in Figure 2.

The 6G is the true enabler of AI for intelligent healthcare, unlike 5G driven smart medical applications. In addition, Virtual/Augmented Reality (VR and AR) are the key indicators for diagnosis in healthcare with high bandwidth and less delay. The main purpose of VR/AR in healthcare is to empower high-resolution multimedia (video, audio, voice) transmission [25,26,27]. One of the limitations of 5G driven VR/AR in healthcare for multimedia transmission is that it does not support the real-time platform to end-users [26]. Thus, it is vital to validate the fuzzy-based sustainable, interoperable, and reliable algorithm (FISRA) with appropriate parameters in e-healthcare applications. The 6G is one of the emerging, powerful, and intelligent technologies with high frequency, lower latency, and better connectivity and high stability. Fuzzy-based methods in association with 6G are potential candidates to support 360-degree multimedia transmission at edge nodes with high efficiency and better quality, which is not supported by 5G driven networks and conventional healthcare trends [28]. 

In addition, joint AI and 6G methods can easily adopt the VR/AR features for smooth 360-degree video streaming at edge nodes with high packet delivery, seamless connectivity, interoperability, and convergence. The 6G always promotes highly interactive and novel video-coding techniques for 360-degree on-demand and live video delivery at a faster pace through edge devices, which is very vital for VR/AR driven healthcare applications. Due to a rapid rise in the use of mobile edge computing and edge caching approaches and trends, latency and load at the backhaul is distributed fairly among end-users with the help of 6G, where 5G does not reveal satisfactory results [29].

The four key contributions of this research are presented as follows. 

First, the development of a single chip-based wearable electrocardiogram (ECG) by adopting analogue front end (AFE) chip model ADS1292R for collecting the electrocardiogram (ECG) data to examine the health status of elderly and emergency patients. Additionally, healthcare monitoring is done through the IoT-based CPS.

Second, the proposal of a fuzzy-based sustainable, interoperable, and reliable algorithm (FSIRA), which is an intelligent and self-adaptive decision-making approach for connected healthcare. Our proposed FSIRA is helpful for physicians to choose the more relevant and highly prioritized indicators, for example, reliability in relation to packet loss ratio, convergence in mapping with delay, and interoperability in trade-off with throughput for enhancing the health quality of the patients at economical rates. 

Third, a cloud-based decisive block diagram and 6G framework are proposed for the connected healthcare in the presence of IoT driven CPS.

Fourth, the relationship between reliability in relation to packet loss ratio, convergence in mapping with delay, and interoperability in association with throughput is established for IoMT driven connected healthcare. CPS is the key system to properly handle and monitor the healthcare platform.

The rest of the paper comprises various sections. Section 2 rigorously reviews the vast literature. Section 3 presents the methodology, block diagram, framework and proposed FSIRA by considering requirements of the healthcare. Section 4 reveals the experimental analysis by mapping three critical parameters, i.e., reliability, convergence and interoperability with PLR, delay, and throughput, accordingly. Finally, the conclusion and future research are presented in Section 5.

## 2. Existing Works

IoT and CPS are the interdependent entities for smart and pervasive systems. Recently, several research works on energy efficient IoT, medical healthcare, WSN, and WBANs/WBSNs were presented, but there are still large gaps in the research to be filled. Some of these works are reviewed one by one. Authors in [1,2] propose novel transmission power control, energy harvesting and body-posture-based energy saving algorithms for smart healthcare in WBAN, but power management is not considered. Researchers in [3,4] examine several power and battery efficient techniques for media transmission, but their research is oversimplified to talk about power management schemes. The work of [5,6,7] discusses the design of the aggregated energy scheduling approach based on the routing mechanism, but the power control and management methods are totally oversimplified. The authors of [8,9,10] present the resource-allotment and TPC techniques for energy saving. Researchers in [11,12] designed the medium access control and frequency selection based on the resource management algorithm and frameworks, but they did not consider the innovative techniques for power management in medical applications. Authors in [13] present the energy harvesting power allocation and cluster-based energy saving techniques for power for BSN and WSN, respectively, but power management is not the center of attention. 

Authors in [14] developed the novel QoS-oriented algorithms by adopting the solar energy harvesting idea in BSN, but the power management from different aspects is not the central point. Researchers in [15] developed the integrated power and energy harvesting technique for fair resource allocation in BSN and healthcare, but they do not address the proper power saving in the BSN. Authors in [16] developed the hybrid transmission control and battery charge-aware schemes; however, their work does not address the CPS driven e-healthcare application. Researchers in [17] developed duty-cycle management-based charge optimization in BSN, but their work is oversimplified to present the power management algorithms for green and smart healthcare. Researchers in [18,19] designed a TPC-based technique for energy optimization, and dynamic power control methods for WSNs and wireless networks, respectively. Authors in [20,21,22,23,24] examined and optimized the impact of the energy of TPC and the lifetime of WSNs and WPT systems, respectively, while they do not concentrate on the power management schemes in BSNs. The authors of [30,31,32] developed the resource allocation, TPC-based techniques for the cognitive radio, and wireless power transfer networks, but their work does not focus on the power management for smart healthcare. Authors in [33,34,35] developed the MAC layer based QoS aware energy efficient technique but do not consider the fair power allocation and management methods. 

Most of the existing research works on 6G focused on the detailed survey and reviews to highlight the need and important limitations and recommendations, but they overlooked developing, testing, and validating the 6G- and AI-based methods and framework [25,26,27,28,29]. Thus, this gap is filled by our conducted research by proposing a 6G-based intelligent resource allocation approach at the fog nodes for healthcare applications. 

To remedy the barrier of time and space requirements, the unavailability of real-time Telesurgery, and holographic communication (Virtual Reality, Augmented Reality and Mixed Reality) in 5G, it is of dire need to think of 6G as a future vision and paradigm shift with the slogan, ‘intelligent and cost-effective healthcare for all at anywhere’. Moreover, due to the ongoing requirement of intensive care and facilities of elderly services at faster rates it is of vital importance to adopt intelligent 6G technology, because 5G is found as less promising and unsatisfactory. 

Authors in [25] developed the energy efficient algorithms and ECG data collection platform, then validated these over both hardware and software by collecting real-time ECG data of patients. Their work is a potential guideline for future research in healthcare. In [26], green and reliable techniques were developed for vehicular communication over 5G networks. In addition, they validated the results by setting the 5G testbed. Researchers in [27] present the detailed survey on the 6G vision for healthcare up to 2030, and significance, challenges, limitations, and potential solutions are presented. An edge-computing-driven data detection mechanism for traffic light monitoring is proposed at the user side in [28]. A detailed review on the 6G opportunities and challenges is presented in healthcare in [29]. 

## 3. Materials and Methods

### 3.1. Wearable Platform for ECG Collection 

Wearable devices are the key enablers for connected healthcare to examine and monitor emergency and elderly patients at any time. A wearable ECG single-led chip was designed in association with an analogue front end (AFE) chip, i.e., ADS1292R. Continuous ECG collecting and monitoring channel is possessed by that chip for emergency elderly patients in the connected healthcare platform, in addition to several other key entities. For instance, wireless micro control unit (MCU) of CC2540F256 and BLE with a data rate of 1 Mbps are presented, as depicted in Figure 1 and Figure 2. Two 24-bit delta-sigma analogue to digital converters as programmable gain amplifiers were adopted for proper monitoring and examination of the collected ECG data. In addition, sampling rates of ADCs from 125 SPS to 8000 SPS with proper monitoring of the digital data were selected through serial peripheral interface (SPI).

### 3.2. Proposed Fuzzy-Based Sustainable, Interoperable, and Reliable Algorithm

In this section we propose the fuzzy-based sustainable, interoperable, and reliable algorithm (FSIRA) which is an intelligent and decision-making algorithm for maximizing the CPS-based healthcare operation time with high reliability, interoperability, and convergence.

#### 3.2.1. Joint Interoperability and Convergence Platform for CPS Based Connected Healthcare

**Figure 3:**
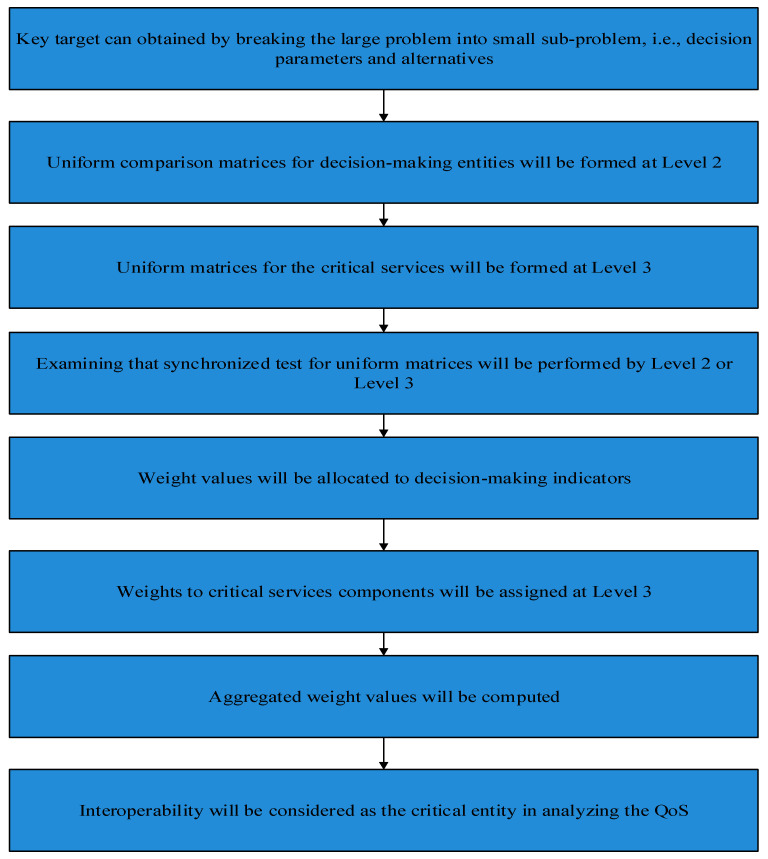
Flowchart of the proposed FSIRA for CPS based connected healthcare.

In this section we propose the sustainable cloud-based joint convergence and interoperability block diagram and framework for the regular monitoring and service provisioning for emergency and elderly patients in the CPS driven connected healthcare as shown in Figure 1 and Figure 2, accordingly. Convergence means interconnecting the things so communication can occur, but interoperability is the use of standard, complementary communications technologies and processes that ensure machines produced by different manufacturers and organizations can speak the same language as defined by the healthcare world, as shown in Figure 3.

In sensor-based wearable devices, CPS has resulted in resounding improvements in facilitating healthcare sectors at reasonable costs, ease, and comfort. In other words, it can be said that CPS is the paradigm shift in the medical market. Therefore, due to the massive IoT driven devices from every corner of the market, a connection is established between gateway and other network entities [1]. For example, a perfect connected healthcare system is made of various devices such as sensors, actuators, RFID tags, accelerometers, etc. The fuzzy-based decision-making technique is the most promising and prominent mechanism that has been incorporated in various fields, for example, healthcare, academia, and industry, etc. The key purpose is to select the appropriate network metric at the Level 1, then to put the decision-making parameters at the middle or Level 2 and decision-making entities at the third or last level.

**Figure 4:**
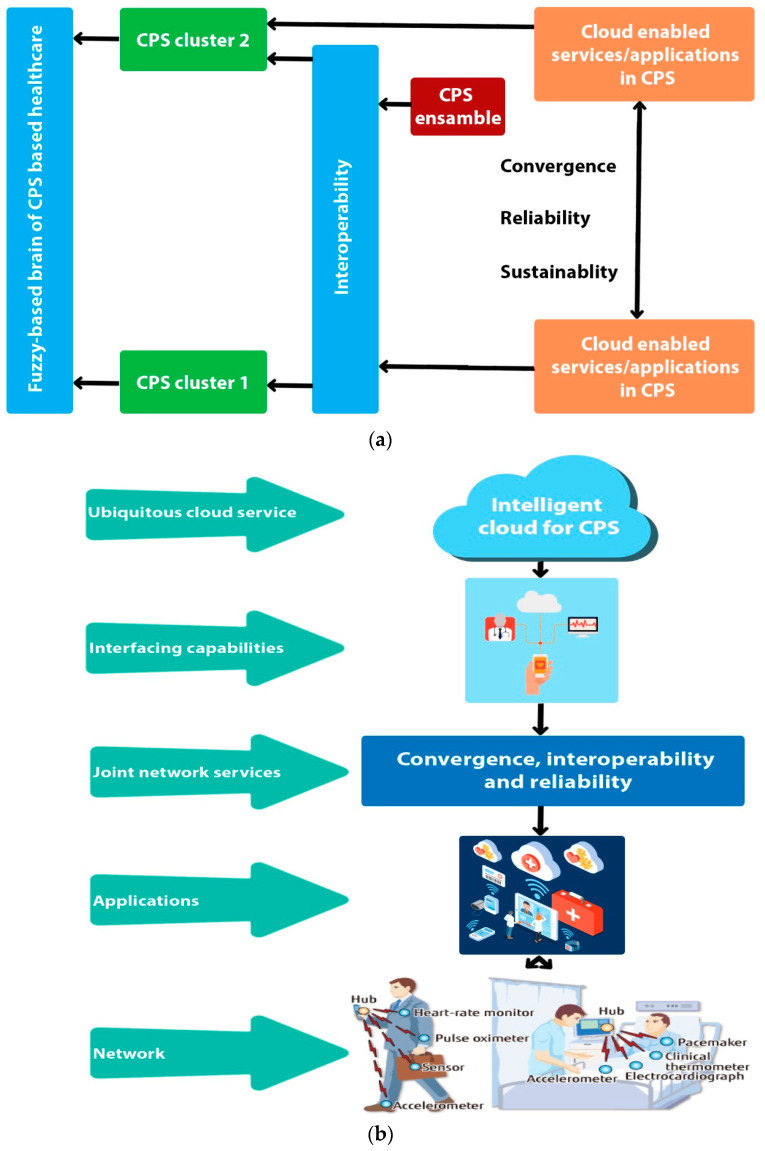
Proposed joint convergent, interoperable, and reliable healthcare: (**a**) block diagram, (**b**) 6G framework.

**Figure 5:**
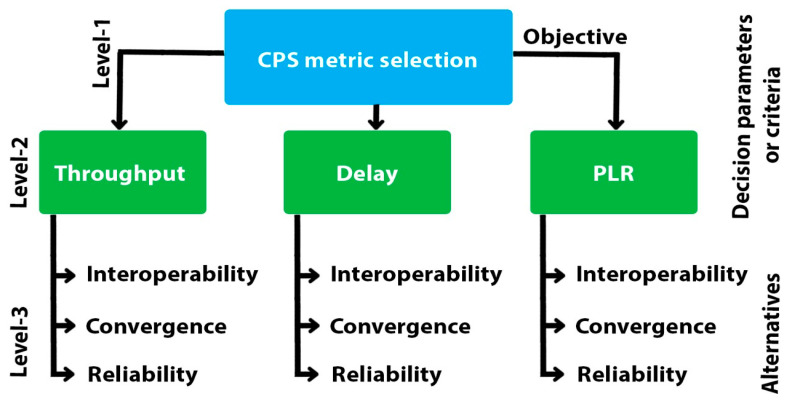
Fuzzy-based entity selection in IoT-based smart healthcare.

Figure 4 and Figure 5 depict the systematic method of critical performance parameter selection. Following, main target-achieving ingredients are compared to obtain the standard level of importance by adopting Table 1. Different levels of importance such as equal, moderate, strong, and very strong are used, while selecting the critical performance indicator allotted with specific values such as, 1, 3, 5, 7 and 9, accordingly, while the loss compensation values are represented by 2, 4, 6 and 8. Mostly small units are considered for making larger values as their multiple. In the last step, the fuzzy-based matrix directs the composite weights to a decisive entity. On the basis of that rule, we assigned the value 1 to throughput and allocate values from 1 to 9 to delay. Again, to prioritize one entity over other, there are more specific description such as the 1_1 value, which highlights that both throughput and delay treated in a similar way, while 1_2 reveals importance of throughput two times more than the delay. Thus, various yardsticks are set from 1_1 to 1_9 to assign the specific weight or importance to the decision-making factors in accordance with services as shown in Table 1. 

**Figure 6:**
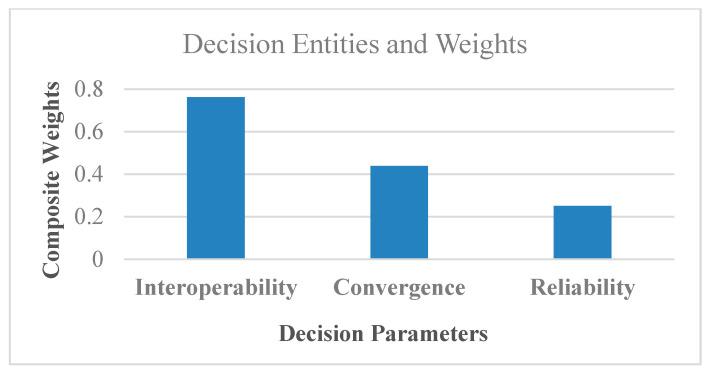
Performance analysis of CPS in connected healthcare.

**Figure 7:**
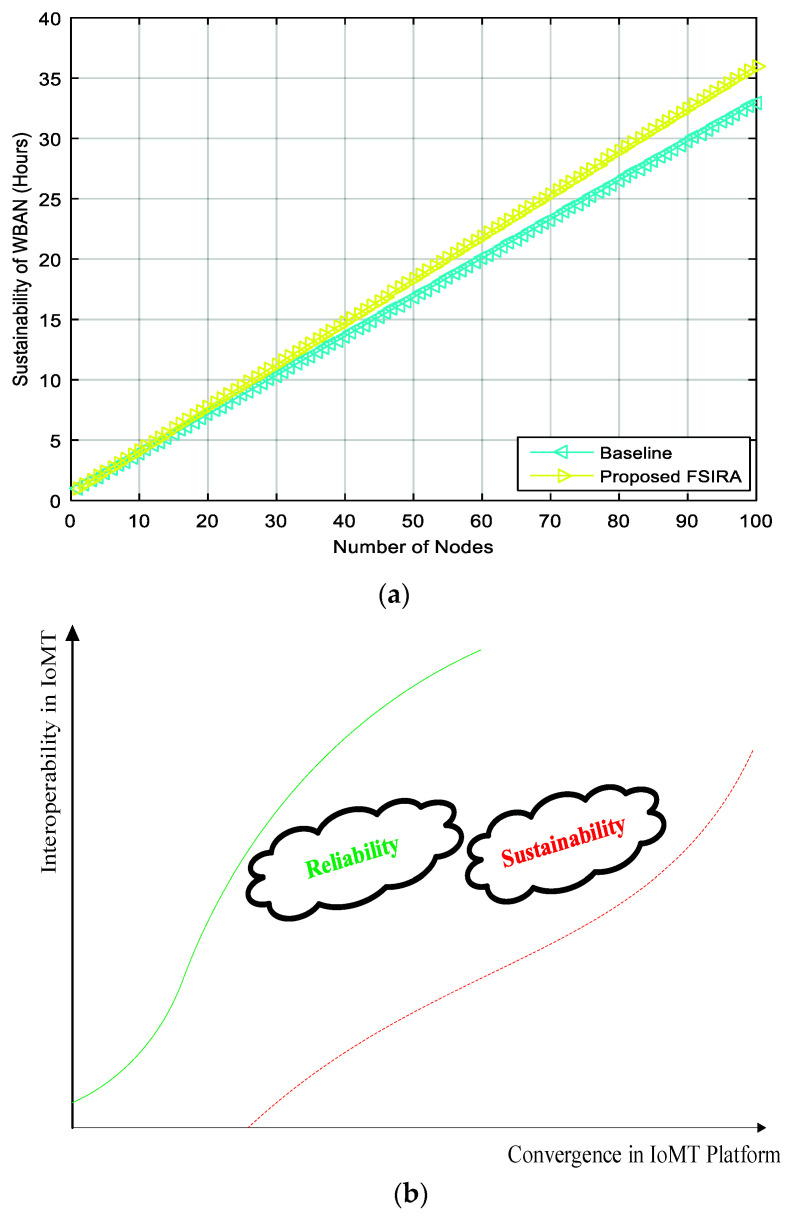
Relationship between (**a**) number of nodes and sustainability, (**b**) convergence and interoperability.

The fuzzy-enabled technique systematically arranges the critical challenge into several sub-problems in different levels. In addition, its procedural mechanism is defined and classified in the particular goal, options and levels in decision-making entities as represented in Figure 6. Assume n factors from $a_{1},\;\ldots ,\;a_{n}$ are compared which indicate the importance of one parameter, i.e., $a_{i}$ in association with $a_{j}$ by $a_{ij}$ and builds the square matrix $M=(a_{ij})$ of order n with the condition of $a_{ij}=1/a_{ji}$, for i≠j, and $a_{ii}=1$, known as the complementary matrix. Transition between weights directs towards the consistency as, $a_{ik}=a_{ij}\times a_{jk}$ for all i, j and k. If $a_{ij}$ is computed properly with the help of measured datasets than such matrix can be formed. After that, vector V of order n forms the matrix such as MV=λ×V, in which V and λ express the eigenvector and eigenvalue accordingly. For further explanation see the proposed FSIRA in Figure 7.

In order to achieve the uniform matrix, there must be condition λ=n, while on the other hand a matrix built by human perception and decision-making ability follows the criteria $a_{ik}=a_{ij}\times a_{jk}$, which in practice cannot be achieved due to various modes and lack of persistency in the decision-making ability. In that condition, V vector fulfils the condition such as $MV=\lambda_{\max}\times V\geq n$. Contradiction in human thinking and perspective is obtained by examining the error difference between $\lambda_{\max}$ and n. However, if $\lambda_{\max}=n$ the static or steady state of the entire matrix is achieved. In the last step we compute the consistency index (CI) with the support of expression $\left(\lambda_{\max}-n)/(n-1\right)$, which needs to be examined and evaluated against random experience. To portray the priority and importance level of decision-making entities it is better to form large data samples with uniform and persistent style in random patterns by adopting the consistency index (CI).

The key consistency ratio (CR) can be obtained by dividing computed CI with the relevant random matrix’s index value. It is examined and observed that if CR gets larger than 0.1 than human perception will be misaligned and non-reliable, but rarely CR values larger than 0.1 can be considered. On the contrary, a zero CR value gives the exact and synchronized match to the human judgment. The proposed FSIRA is very promising and decisive to break the big problem into several sub-problems and systematically resolves the entire complex situation based on specific critical values and decision-making factors. An intelligent and self-adaptive fuzzy-enabled algorithm is proposed to achieve the sustainable, interoperable, reliable, and connected IoT driven healthcare platform by properly selecting the critical parameters and decision-making ingredients. This section comprises various sub-sections each of them is described one by one.

##### 3.2.2. Adaptive and Decisive Process 

The operation/working time is efficiently utilized while obtaining the sustainable, interoperable, and reliable IoMT-based connected healthcare. A more systematic procedure is built at the initial level as presented in Figure 6 and Figure 7. The key 6G network performance indicators, for instance, throughput, delay, and PLR, playing the role of decision-making entities and interoperability, convergence and reliability, are considered as the set of alternatives/options in CPS, arranged at Level 2 and Level 3 of the strategy accordingly.

##### 3.2.3. Similarity Matrix 

These types of matrixes are classified into rows and columns, in addition to numerous parameters included with set of weights from 1 to 9 as presented in Table 1. We assumed 1 as the cross-sectional value from top-left to bottom-right sides. At the start, the triangular matrix at the top-level is filled by prioritizing the row vector A and coordinated column vector B, as represented in Table 1. In the last lower side, a triangular matrix can be formed by filling the upper triangular matrix and considering Equation (1).

(1) $$a_{ji}=\frac{1}{a_{ij}}$$

The main uniform and pair-based matrix containing ith row and jth column $a_{ji}$ is achieved, as correlated and similarity matrices for decision-making entities can be drawn at Level 2 and Level 3, respectively.

$$M=\begin{array}{c} Interoperability\\ Convergence\\ Reliability \end{array}\left[\begin{array}{ccc} Interoperability & Convergence & Reliability\\ 1 & 2/1 & 3/1\\ 1/2 & 1 & 2/1\\ 1/3 & 1/2 & 1 \end{array}\right]$$

## 4. Experimental Setup

This section develops the experimental platform for connected healthcare by developing a fuzzy-based sustainable, interoperable, and reliable algorithm (FISRA) by categorizing the results and evaluation parts accordingly for emergency and elderly patients. Real-time data are collected by attaching the ECG chip on the human body while resting and driving a bike, because in both conditions heart rate varies. 

### 4.1. Results

We performed our experiment by considering 20 real subjects (10 male and 10 female) of age between 35 to 60 years after analyzing their heart attacks and other diseases. A fuzzy-based decisive and intelligent algorithm named FISRA is proposed to optimize the QoS in CPS-based connected healthcare by choosing the important priority and decision-making parameters. 

Step-1: Calculating pair-wise matrix.

$$M=\begin{array}{c} Interoperability\\ Convergence\\ Reliability \end{array}\left[\begin{array}{ccc} Interoperability & Convergence & Reliability\\ 1 & 2/1 & 3/1\\ 1/2 & 1 & 2/1\\ 1/3 & 1/2 & 1 \end{array}\right]$$

Or, Matrix M can be presented as:

$$M=\left[\begin{array}{ccc} 1.0 & 2.0 & 3.0\\ 0.5 & 1.0 & 2.0\\ 0.3333 & 0.5 & 1.0 \end{array}\right]$$

Step-2: By squaring Matrix M.

$$s\left[\begin{array}{ccc} 1.0 & 2.0 & 3.0\\ 0.5 & 1.0 & 2.0\\ 0.3333 & 0.5 & 1.0 \end{array}\right]\left[\begin{array}{ccc} 1.0 & 2.0 & 3.0\\ 0.5 & 1.0 & 2.0\\ 0.3333 & 0.5 & 1.0 \end{array}\right]=\left[\begin{array}{ccc} 3.0 & 5.5 & 10\\ 1.6666 & 3.0 & 5.5\\ 0.9166 & 1.6666 & 3.0 \end{array}\right]$$

Step-3: Calculating eigenvector.

$$M=\left[\begin{array}{ccc} 3.0+ & 5.5+ & 10\\ 1.6666+ & 3.0+ & 5.5\\ 0.9166+ & 1.6666+ & 3.0 \end{array}\right]$$

$$M=\frac{SoR\left[\begin{array}{c} 18.5\\ 10.1666\\ 5.5832 \end{array}\right]}{SoR\_total\;34.2498}$$

$$\left[\begin{array}{c} 18.5/34.2498\\ 10.1666/34.2498\\ 5.5832/34.2498 \end{array}\right]=\left[\begin{array}{c} 0.5401\\ 0.2968\\ 0.1630 \end{array}\right]\to \mathrm{Eigenvector}=E0$$

$$\frac{\left[\begin{array}{c} 0.5401\\ 0.2968\\ 0.1630 \end{array}\right]}{1.0}$$

Again Step-1: Generating pair-based square matrix.

$$M=\left[\begin{array}{ccc} 3.0 & 5.5 & 10\\ 1.6666 & 3.0 & 5.5\\ 0.9166 & 1.6666 & 3.0 \end{array}\right]$$

Again Step-2: Squaring the matrix.

$$M=\left[\begin{array}{ccc} 3.0 & 5.5 & 10\\ 1.6666 & 3.0 & 5.5\\ 0.9166 & 1.6666 & 3.0 \end{array}\right]\left[\begin{array}{ccc} 3.0 & 5.5 & 10\\ 1.6666 & 3.0 & 5.5\\ 0.9166 & 1.6666 & 3.0 \end{array}\right]=\left[\begin{array}{ccc} 27.3326 & 49.666 & 90.25\\ 15.0409 & 27.3326 & 49.666\\ 8.2772 & 15.0409 & 27.3323 \end{array}\right]$$

Again Step-3: Calculating eigenvector.

Eigenvector E1 or V efficiently calculates relevant eigenvalue ($\lambda_{\max}$), consistency index, RI and consistency index to obtain the decisive entities in Level 2.

$$\left[\begin{array}{ccc} 27.3326+ & 49.666+ & 90.25\\ 15.0409+ & 27.3326+ & 49.666\\ 8.2772+ & 15.0409+ & 27.3323 \end{array}\right]=\left[\begin{array}{c} 167.2486\\ 92.0395\\ 50.6504 \end{array}\right]=\left[\begin{array}{c} 0.5396\\ 0.2969\\ 0.1634 \end{array}\right]\to \mathrm{Eigenvector}=E1$$

$$\frac{\left[\begin{array}{c} 0.5396\\ 0.2969\\ 0.1634 \end{array}\right]}{1.0}$$

$$D=E1-E0=\left[\begin{array}{c} 0.5396\\ 0.2969\\ 0.1634 \end{array}\right]-\left[\begin{array}{c} 0.5401\\ 0.2968\\ 0.1630 \end{array}\right]=\left[\begin{array}{c} -0.0005\\ 0.0001\\ 0.0004 \end{array}\right]\to \mathrm{Almost}\;\mathrm{zero}$$

Step-4: To compute consistency index (CI) by using $MV=\lambda_{\max}\times V$, whereas, M,V and $\lambda_{\max}$ define the matrix, eigenvector and maximum eigenvalue, accordingly.

$$\left[\begin{array}{ccc} 1.0 & 2.0 & 3.0\\ 0.5 & 1.0 & 2.0\\ 0.3333 & 0.5 & 1.0 \end{array}\right]\left[\begin{array}{c} 0.5396\\ 0.2969\\ 0.1634 \end{array}\right]=\lambda \;\max\left[\begin{array}{c} 0.5396\\ 0.2969\\ 0.1634 \end{array}\right]$$

$$\left[\begin{array}{ccc} 0.5396+ & 0.5938+ & 0.4902\\ 0.2698+ & 0.2969+ & 0.3268\\ 0.1798+ & 0.1484+ & 0.1634 \end{array}\right]=\lambda \;\max\left[\begin{array}{c} 0.5396\\ 0.2969\\ 0.1634 \end{array}\right]$$

$$\left[\begin{array}{c} 1.6236\\ 0.8935\\ 0.4916 \end{array}\right]=\lambda \;\max\left[\begin{array}{c} 0.5396\\ 0.2969\\ 0.1634 \end{array}\right],\;\lambda \;\max=\left[\begin{array}{c} 1.6236/0.5396\\ 0.8935/0.2969\\ 0.4916/0.1634 \end{array}\right]=\left[\begin{array}{c} 3.0088\\ 3.0094\\ 3.0085 \end{array}\right]$$

The aggregated maximum eigenvalues at n = 3 is calculated as follows.

(2) $$\lambda_{\max}=\frac{\begin{array}{ccc} 3.0088+ & 3.0094+ & 3.0085 \end{array}}{3}=\frac{9.0262}{3}=3.0087$$

Now, from Equation (3) we have

(3) $$CI=\frac{\lambda_{\max}-n}{n-1}=\frac{3.0087-3}{3-1}=\frac{0.0087}{2}=0.0043<0.1$$

(4) $$CR=\frac{CI}{RI}=\frac{0.0043}{0.58}=0.007413$$

At *CI* value 0.0043 < 0.1, uniform and adaptive pair-wise comparison matrices are formed. The consistency ratio (*CR*) can be achieved by following Equation (4) and Table 1 (RI = 0.58 at n = 3).

At *CR* < 0.1, evaluation can be persistent and uniform.

Interoperability = 0.761 → first highly prioritized parameter for IoMT driven connected healthcare.

Convergence = 0.438 → second prioritized element for connected healthcare. 

Reliability = 0.251 → third and less prioritized element for connected healthcare. 

### 4.2. Evaluation

This sub-section evaluates the performance of the CPS-based smart healthcare by optimizing the QoS with the support of fuzzy-based sustainable, interoperable, and reliable algorithm (FISRA) with decisive and intelligent features. Such self-adaptive approaches integrate the various technologies to make the system sustainable, reliable, and self-driven. The fuzzy-oriented intelligent features comparison matrix is organized into pairs then composite weights are categorized at Level 2, and in the last step, main industrial entities are achieved. The proposed FSIRA evaluates and examines the sustainable, interoperable, reliable and CPS driven connected healthcare as the key target with decisive, competitive, and prioritizing nature.

**Figure 8:**
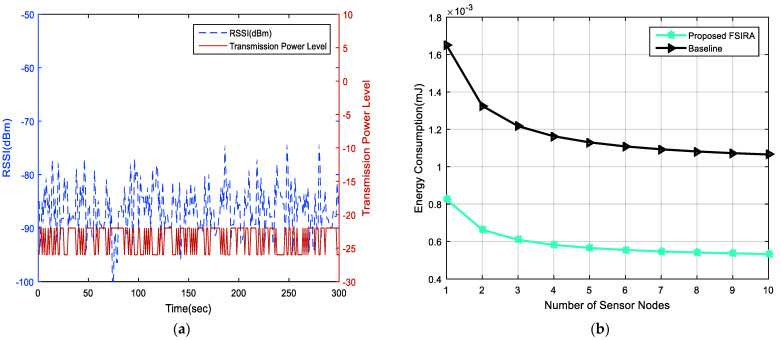
Analysis of (**a**) reliability, (**b**) convergence in healthcare system.

In addition, the linear connections between decision parameters and the aggregate weights are obtained, as shown in Figure 6, while the interlink between nodes and sustainability (i.e., lifetime) is found, as shown in Figure 7a for baseline and proposed FSIRA for the CPS driven connected healthcare. It is observed that the proposed FISRA shows higher sustainability than the baseline, thus is the potential candidate for connected healthcare platform. The linear and exponentially increasing relationship is found between the main components of the CPS, for example, convergence and the interoperability to effectively analyze the overall impact of connected healthcare on sustainability. Hence, we can say that convergence, interoperability, reliability, and sustainability are the inter-dependent entities in getting less error as well as maximum output-based CPS as presented in Figure 7b. The relationship between time, RSSI and transmission power control is revealed in Figure 8a. It is analyzed and examined that RSSI gives insight about the quality of the received signals while energy efficiency level is highlighted by the transmission power control. Figure 8b addresses the trade-off between energy optimization and number of sensor nodes, and it is evaluated that more energy is saved with an increased number of nodes by the proposed FSIRA and less by conventional techniques such as baseline. Due to a large number of sensor-based devices, efficiency is distributed in the entire network proposed. The fuzzy-enabled method shows greater efficiency than the baseline. In addition, we evaluated the overall performance of the fuzzy-enabled CPS by analyzing the rate of duty-cycle variation in the healthcare. 

### 4.3. Validity of Results 

Our proposed algorithm and entire experimental results are validated by considering the joint CPS, 6G and AI features for achieving the sustainable, interoperable, convergent, reliable, connected, and cost-effective healthcare in MATLAB. Table 2 shows the key parameters that were studied, such as RSSI (highest −80 dBm, lowest −100 dBm), transmission power (−20 dBm), modulation level in bits 256QAM, transmission rate (6 Mbps), deviation in RSSI (7 dB), Channel Bandwidth (10 MHz), maximum transmission power (25 mW), total time (1560 s), average distance between CPS nodes (2 m), and reliability (95%). Finally, we provide simulation and experimental results [28,31,32] to validate the effectiveness of proposed FISRA with possible future recommendations. To validate the packet loss probability (around 10^−5^–10^−7^ in 6G) of an IoT device, the system needs to send 100–109 packets.

Still, 5G needs high maturity to remedy the real-time tactile feedback issues in the existing healthcare applications and maximum QoS in terms of availability, i.e., 99.999% reliability, ultralow latency, micro-mili s, and high mobility and 360-degree coverage. In addition, time and space requirements in remote surgery with a higher guarantee of better workflow in healthcare are the crucial problems to be solved by 6G with outstanding and revolutionary performance [33].

## 5. Conclusions and Future Research

This Artificial intelligence (AI)-based self-adaptive edge-based healthcare platform has become the paradigm shift in facilitating the medical world with the help of emerging sensor-based technologies, which comprises human activities and their personal devices through internet proxies. Thus, this paper proposes the fuzzy-based intelligent decision-making technique for selecting the most critical parameter from the pool of the performance indicators in CPS. In addition, the joint convergence and interoperability framework is proposed to select the appropriate and urgent metrics for enhancing the performance of CPS with minimum cost and maximum profit strategy. Moreover, there are three main contributions: first, the development of the hardware platform for ECG data collection from 20 subjects (i.e., emergency and elderly). Second, the proposal of a fuzzy-based sustainable, interoperable, and reliable algorithm (FSIRA), which is intelligent decision-making for the CPS driven connected healthcare. The fuzzy-based algorithm is appropriate for physicians to choose more appropriate and high priority entities, for instance, reliability in relation to with packet loss ratio, convergence in trade-off with delay, and interoperability in association with throughput for future medical markets. Third, a cloud-based CPS driven connected healthcare platform (block diagram and framework) is proposed. Fourth, the relationship between network metrics (throughput, delay, and PLR) and the performance of the CPS (reliability, convergence, and interoperability) is established for the CPS-based connected healthcare.

The following are the few limitations of the proposed method.

- More computational and processing complexity is observed while dealing with large datasets, due to the resource-constrained features of the IoT-based CPS device’s developed testbed.
- While establishing uniform standard for CPS based heterogenous healthcare, it is vital to analyze the interconnection between performance indicators such as convergence, delay, and interoperability, but it consumes more power and hence the charge drain in portable IoT devices.

In near future we are planning to consider more subjects, and 6G with CPS for achieving the cost-effective and efficient medical services to everyone and at any time. In addition, resource allocation mechanism is the dire need for IoT driven healthcare applications.

Highlights of the Manuscripts

- The development of the single chip wearable ECG platform for elderly patient monitoring by adopting CPS.
- The proposal of a fuzzy-based sustainable, interoperable, and reliable algorithm (FSIRA) for CPS-based connected healthcare.
- The proposal of the novel cloud-based 6G framework for CPS-based connected healthcare.
- The establishment of the relationship between reliability, PLR, convergence, delay, interoperability, and throughput for the CPS-based smart healthcare platform.

## Figures and Tables

**Table 1:** Parameter selection with fuzzy rule.

Comparison	Value of *a_ij_*
A and B have same priority	1
B has a higher priority than A	3
A has higher priority than B	5
A has a higher priority than B	7
A is essential unlike B	9
Correlation among all entities	2, 4, 6, 8
Correlation for compensating loss factor	1/3, 1/5, 1/7, 1/9

**Table 2:** Experimental parameters.

Parameter	Specifications
Number of IoT nodes	10
Cell layout	Hexagonal
Threshold RSSI	−85 dBm
Standard deviation (σ)	5 dBm
Duty-cycle	1%
Carrier frequency	30 GHz
6G Bandwidth	60 GHz
TP levels	{−6, −5, −4, −3, 2, 1, 0, 1, 2, 3, 4, 5, 6}
eNodeB Tx power	45 dBm
Fading model	Rayleigh
Channel model	CDL-D
Operation time (T)	4 min
Delay	1 ms
Data packet length	300 bytes
Data packet interval	50 s
Data Rate	1 Gbps
Noise figure	8 dB
Noise PSD	−175 dBm/Hz
Traffic model	2 Mbps (adaptive)
Processing delay	0.1 ms
